# One Iron Injection Is Not Enough—Iron Status and Growth of Suckling Piglets on an Organic Farm

**DOI:** 10.3390/ani9090651

**Published:** 2019-09-04

**Authors:** Katharina Heidbüchel, Jessica Raabe, Lisa Baldinger, Werner Hagmüller, Ralf Bussemas

**Affiliations:** 1 Institute of Organic Farming, Johann Heinrich von Thünen Institute, Trenthorst 32, 23847 Westerau, Germany (J.R.) (L.B.) (R.B.); 2 Institute of Organic Farming and Farm Animal Biodiversity, Austrian Research and Education Center Raumberg-Gumpenstein, 4600 Thalheim/Wels, Austria

**Keywords:** pig, organic agriculture, iron injection, piglet health, iron dextran

## Abstract

**Simple Summary:**

Suckling piglets need more iron for rapid and healthy growth than is available from sow’s milk alone. Therefore, iron supplementation is common on both conventional and organic farms and is usually carried out by a single injection of 200 mg iron during the piglets’ first days of life. However, the long suckling periods in organic pig farming can still lead to iron deficiency, as we found in a study with 288 piglets on an experimental farm. A single subcutaneous injection of iron led to lower iron levels and slower growth until weaning, when compared with giving iron two or three times. We conclude that one iron injection is not enough to ensure adequate iron supply and unimpaired growth of organically reared piglets, and options of additional iron supply need to be explored.

**Abstract:**

Suckling piglets cannot satisfy their demand for the essential trace element iron from sows’ milk alone, which is poor in iron. Therefore, iron is usually supplemented by injecting 200 mg iron (as iron dextran) on day 3 post natum. However, the longer suckling period in organic pig herds leads to iron intake from feed starting later. We hypothesized that one iron injection is not enough for organically reared piglets, and that a second and third injection would improve their iron status and growth. On an organically certified experimental farm, 288 piglets from 32 litters were allocated to three treatments with one iron injection on day 3, two injections on day 3 and 14 and three injections on day 3, 14 and 21 post natum, respectively. One injection resulted in the lowest hematocrit and serum iron levels until day 28 post natum and the lowest daily weight gains until weaning, while piglets receiving two or three iron injections did not differ from each other. Based on these observations, we conclude that one iron injection is not enough to ensure adequate iron supply and unimpaired growth of organically reared piglets, and additional iron supply is necessary.

## 1. Introduction

The most common health disorder in suckling piglets is iron deficiency anemia [1]. Piglets are born with iron reserves of only 35–50 mg [2] and, with a daily demand for 7–16 mg iron [3], these reserves are quickly depleted. The main sources of iron intake in suckling piglets are sows’ milk, feed, and soil. However, intake from sows’ milk covers only 10% of the daily demand [4]. Intake from feed depends on how soon piglets start to eat solid feed. Under the conditions of organic farming and a suckling period of at least 40 days [5], the transition to solid feed happens later than in conventional pig husbandry, where a weaning age of 21 to 28 days is common. When prolonging the suckling period to 63 days, Bussemas and Weissmann [6] found an improved piglet growth rate and a reduced number of medical treatments compared to a suckling period of 40 days. Piglets benefit from longer suckling periods because they can perform their natural behavior in good conditions [1]. On the other hand, this management increased the risk of undersupply with iron. Iron intake from the soil is only possible in outdoor housing systems and, even there, benefits of iron supplementation have been found [7]. Therefore, suckling piglets are routinely supplemented with iron, the intramuscular injection of 200 mg iron in the form of iron dextran on the third day of life being the most common practice [8]. While iron dextran is non-toxic in healthy piglets, there are rare reports of toxicity after intramuscular injection, leading to discussions about alternatives [8]. Subcutaneous injection has been shown to be equally efficient as intramuscular injection [9]. Although there is no information on the rate of iron absorption after subcutaneous injection, Kolb et al. [10] have shown that iron dextran is absorbed more slowly from adipose tissue than from muscle, thereby avoiding excess iron in the circulation. Oral application of iron has been found to be inferior to injection due to lower absorption rates and faster depletion of iron reserves after application [11], but it is of low risk because absorption is left to homeostatic regulation. However, the current regulations for organic farming only allow the application of non-organic iron sources [5]. A study by Hagmüller and Gallnböck [12] showed that oral application of non-organic iron sources achieved unsatisfactory results with organically reared piglets. The only version of oral application that resulted in comparable serum iron levels and body weight gain was an iron dextran paste (day 1 and 7), which is not in accordance with the regulations for organic farming [12]. Several previous studies have focused on improving the iron status of piglets by increasing the iron supply to the sows. Because milk iron content did not increase, effects on the iron status of the piglets failed to appear [11]. However, positive effects on iron content in placenta and litter size have been found by Buffler et al. [13].

With piglet mortality being one of the biggest challenges in organic pig husbandry [14], an adequate iron supply is one of several measures that can be taken to optimize the rearing conditions for piglets. Studies on the iron supplementation of conventionally reared suckling piglets report positive or no effects on iron status, growth and health [15,16,17,18,19]. However, to date, no study under the conditions of organic farming is known. Therefore, we conducted an experiment on an organically certified farm, in which we compared three frequencies of subcutaneous injection of iron dextran. The aim was to assess whether one iron injection is enough to ensure adequate iron supply and unimpaired growth until weaning, and whether giving iron a second and third time improves iron status and weight gain. Preliminary results of this study have been presented at the 14th Wissenschaftstagung Ökologischer Landbau (German Scientific Conference on Organic Agriculture) in 2017 [20].

## 2. Materials and Methods

The experiment was conducted between February and October 2016 on the experimental farm of Thünen Institute of Organic Farming in Trenthorst, Germany. Animal husbandry followed the rules of European Commission Regulation 889/2008 [5]. The study was announced to the Ministry of Energy Turnaround, Agriculture, Environment and Rural Regions of Schleswig-Holstein and was confirmed on 30 March 2016 (V244-27911/2016). The experimental period lasted from the piglets’ third day of life until weaning.

### 2.1. Experimental Design and Animals

The experiment was designed as a completely randomized block design, with the single litter forming one block and the individual piglet as the experimental unit. Only litters consisting of at least 9 healthy piglets of more than 1 kg body weight on day 3 post natum were included in the experiment. In total, the experiment included 32 litters of 23 sows and 288 piglets. Within each litter, on day 3 post natum, nine piglets were randomly assigned to one of the three treatments of iron supplementation, resulting in at least three piglets of each litter receiving the same treatment. Piglets were assigned to treatments by generating a random number between 1 and 3 for each piglet (excel command “randbetween (1,3)”). The three treatments were as follows: Iron1 consisted of a single dose of 200 mg iron dextran on day 3 post natum (3.0 ± 0.35); in treatment Iron2, piglets received iron twice, once on day 3, and again on day 14 post natum (13.7 ± 0.81); piglets assigned to Iron3 received iron three times: on day 3, 14 and 21 post natum (21.0 ± 1.2). Iron supplementation was always carried out by subcutaneous injection of an iron solution equivalent to 200 mg ferrous ions (Fe^3+^) per piglet under the folds of the knee (2 mL Ursoferran^®^, 100 mg mL^−1^, Serumwerk Bernburg AG, Bernburg, Germany).

### 2.2. Housing and Feeding

The piglets were born of crossbred sows (German Landrace × Large White), inseminated with Piétrain. At one week ante partum, sows were moved to individual straw-bedded farrowing pens of 7.8 m^2^ indoor and 5.9 m^2^ outdoor area. Each pen was equipped with a heated creep area for piglets. At two weeks post partum, the sows and their litters were moved to a group suckling unit, in which they stayed in groups of 3 sows plus litters (except one group which consisted of 6 litters) until weaning at day 42 (±1.7). The group suckling system offered 7.8 m^2^ indoor and 3.8 m^2^ outdoor areas per sow and also included a creep area. The rules for grouping the sows were as follows: Piglets´ age differed no more than 5 days; sick sows remained in the individual farrowing pen; no single primiparous sow in a group. Although cross-suckling was not observed, the possibility cannot be completely ruled out because within each group the sows and piglets were free to mix. During gestation, sows were fed the gestation mixture shown in Table 1. Feed was offered twice a day in restricted amounts adjusted to the sows’ body condition, with a minimum of 1.8 kg and a maximum of 4.8 kg per day (as-fed basis). Also, sows had ad libitum access to a grass-clover ley during summer and grass-clover silage during winter. From one week *ante partum* until weaning, sows were fed a lactation mixture (see Table 1) twice a day in restricted amounts adjusted to the litter size and the body condition of the sow up to a maximum of 10 kg per day (as-fed basis). In addition to the lactation mixture, sows received grass-clover silage up to a maximum of 4 kg per animal and day as roughage. From their first day of life on, piglets had access to the sow’s feed. Starting on day 14 post natum, a starter feed was offered ad libitum in the creep area (composition see Table 1). When formulating the diets, recommendations for energy and protein supply were taken from Lindermayer et al. [21], and iron concentration was adjusted according to McDowell [22]. All feeds were produced at the feed mill of the experimental farm and consisted of homegrown cereal grains and grain legumes, supplemented with purchased press cakes and mineral feed (H.Wilhelm Schaumann GmbH, Pinneberg, Germany), citric acid (RFI Food Ingredients, Düsseldorf, Germany), plant oil (Elbmarsch Ölmühle Markt GmbH, Echem, Germany), skimmed milk powder (Gläserne Molkerei GmbH, Dechow, Germany), and a probiotic (Levucell^®^ SB, Lallemand Animal Nutrition, Montreal, Canada, 200 g per 1000 kg feed). 

Male piglets were castrated at the age of 7 days, under inhalation anesthesia with isoflurane (device “Porc Anest 1000”, Promatec Automation AG, Derendingen, Switzerland). In addition, piglets received 2 mg animal^−1^ of the analgesic meloxicam (Metacam^®^, 5 mg mL^−1^, Boehringer Ingelheim Vetmedica, Ingelheim/Rhine, Germany). At the age of 21 days, all piglets were vaccinated against *Mycoplasma hyopneumoniae* (Ingelvac MycoFLEX, Boehringer Ingelheim Vetmedica, Ingelheim/Rhine, Germany) and *Lawsonia intracellularis* (Enterisol^®^ Ileitis, Boehringer Ingelheim Vetmedica, Ingelheim/Rhine, Germany). Both vaccines did not contain any form of iron.

### 2.3. Data Collection

From every feed mixture, one bulk sample was collected and sent to a commercial laboratory for nutrient analysis according to EU regulation EC 152/2009 [24]. Feed consumption of piglet starter feed in the group suckling system was documented per group of 3 litters by documenting feed allowance continually and weighing feed refusals once a week (Table S1), if there were any. Blood samples were taken by puncturing the vena cava cranialis immediately before the iron injections on days 3 (3.0 ± 0.35), 14 (13.7 ± 0.81), 21 (21.0 ± 1.21), and additionally on day 28 post natum (28.1 ± 1.13). One hour after taking the samples, hematocrit in the whole blood was determined by centrifugation for four minutes in a micro-hematocrit centrifuge (model Mikro 12–14, Hettich, 78532 Tuttlingen, Germany). Additional fresh blood samples were centrifugated with 3000 rpm for 15 min at a temperature of 6 °C and then pipetted and stored at −20 °C until analysis at the Austrian Research and Education Center, Institute of Organic Farming and Farm Animal Biodiversity in Thalheim/Wels, Austria. There, a commercial kit was used for colorimetric determination of the iron concentration in serum (cobas^®^ c111 system, Roche Diagnostics GmbH, Mannheim, Germany, 2014). Piglets were weighed individually once a week until weaning, and daily weight gain was calculated from individual body weight. All medical treatments and mortality were documented routinely during the experimental period.

### 2.4. Statistical Analysis

At the start of the experiment, the first three litters were used as a pilot study to estimate the necessary sample size for hematocrit, serum iron and body weight. The estimation followed the method described by Littell et al. [25] and was carried out using SAS [26]. The model was Y = treatment_k_ + day_l_ + treatment_k_ × day_l_ + litter_m_ + piglet_n_(treatment_k_) (legend see below). For a dataset of 30 litters (=270 piglets), the expected power was estimated at 0.95–0.97 for the effect of group, 1.00 for the effect of day and 0.70–0.96 for treatment × day. The final dataset included 32 litters.

Statistical analysis was performed with SAS^®^ software [26]. Using PROC MIXED and the covariance structure “variance components”, the following model was used for the analysis of the covariance of hematocrit, serum iron and body weight:

Y_klmno_ = μ + treatment_k_ + day_l_ + treatment_k_ × day_l_ + litter_m_ + piglet_n_(treatment_k_) + b_1_ × weight_day3 + ε_klmno_

where μ = overall mean; treatment_k_ = fixed effect of treatment (k = Iron1, Iron2, Iron3); day_l_ = fixed effect of day of life (l = 3, 14, 21, 28, 42); treatment × day = interaction between treatment and day; litter_m_ = random effect of litter (m = individual number of the litter); piglet_n_(treatment_k_) = random effect of piglet (n = individual number of the piglet) within treatment; b_1_ = regression coefficient of birth weight; weight_day3 = continuous linear effect of the live weight of piglet on the third day of life; ε = residual error.

The effect of sex was initially included in the model but dropped due to a lack of significance. When analyzing daily weight gain until weaning, the effect of day and the interaction treatment × day were dropped from the model. For multiple comparisons of means, the Tukey test was used. Probability values <0.05 were interpreted as representing statistically significant differences. Tables 2–4 show least square estimates of the interaction treatment × day, the standard errors of the means (SEM) and *p* values of the effects of treatment, day and treatment × day. Values within a row with different lowercase superscripts differ significantly at *p* < 0.05. Values within a column with different capital superscripts differ significantly at *p* < 0.05. Due to low incidences of medical treatments and mortalities during the experimental period, no statistical analysis was done. Instead, the numbers of piglets needing medical treatment and the numbers of lost piglets are given in Table 5.

## 3. Results

### 3.1. Blood Parameters

The effects of different frequencies of iron supplementation on hematocrit in piglet blood are shown in Table 2. On days 3 and 14 post natum, no differences between treatments were found. On day 21 and 28 post natum, hematocrit in the blood of Iron1 piglets was significantly lower than after two or three injections. Irrespective of treatment, hematocrit content was significantly higher on day 14 than on day 3. In Iron1, hematocrit did not differ between day 14 and 21, but was significantly lower on day 28 than on days 14 and 21. Both in Iron2 and Iron3, hematocrit was significantly higher on day 21 than on day 14, but the difference between day 21 and 28 was only significant in Iron3. Serum iron content showed almost the same pattern as hematocrit (Table 3): no differences were observed on day 3 and 14, but on day 21 Iron1 resulted in serum iron being significantly lower than in Iron2 and Iron3. On day 28, serum iron was significantly lowest in piglets assigned to Iron1 and significantly highest in Iron3, with the value being more than four times higher in Iron3 than in Iron1. In Iron1, serum iron content on day 14 was significantly higher than on all other days, which did not differ from each other. In treatments Iron2 and Iron3, serum iron content significantly increased until day 21, while days 21 and 28 did not differ.

### 3.2. Performance and Health of Piglets.

**Figure 1:**
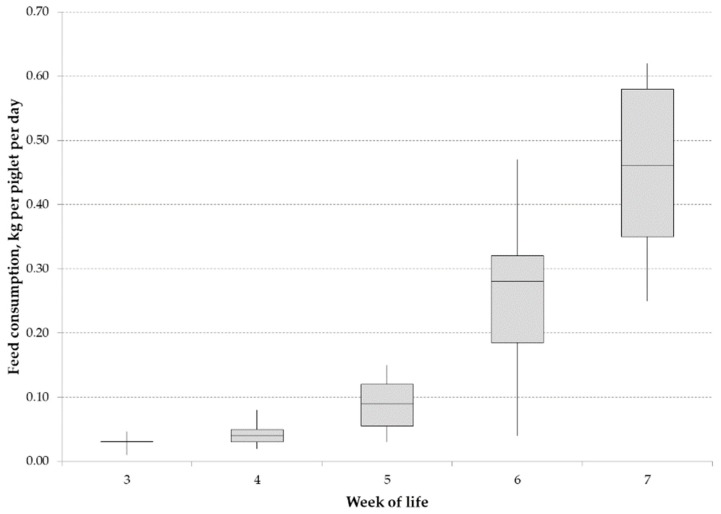
Feed consumption of piglets receiving different frequencies of iron supplementation. The box-plots show minimum, first quartile, median, upper quartile and maximum values, and the values are given on an as-fed basis.

Due to the experimental design (all treatments within each litter), feed consumption of piglet starter feed could not be differentiated between treatments and was documented as the sum consumed by all piglets of a group in the group suckling system. Figure 1 shows the feed consumption throughout the suckling period.

Although the frequency of iron supplementation did not influence the body weight of piglets until the age of 28 days, both body weight at weaning and daily weight gain until weaning were significantly affected (Table 4). While not differing from each other, Iron2 and Iron3 resulted in significantly higher daily weight gain and significantly higher body weight at weaning than Iron1. Neither medical treatments nor animal mortality throughout the experimental period showed considerable differences between the treatments (Table 5). In total, medical treatments were necessary for 10.4% of the piglets assigned to Iron1, 9.0% of piglets in Iron2, and 7.5% of piglets in Iron3. Animal mortality amounted to 3.1%, 3.0%, and 5.4% of piglets in Iron1, Iron2 and Iron3 treatments, respectively.

## 4. Discussion

This study was conducted in order to assess whether one subcutaneous iron injection (200 mg iron as iron dextran) is enough to ensure adequate iron supply and unimpaired growth of suckling piglets on an organic farm, and whether administering iron two or three times improves iron status and weight gain. In total, 32 litters of at least nine healthy piglets of more than 1 kg body weight on day 3 post natum participated in the study. Within each litter, all three treatments were tested, resulting in at least three piglets of each litter receiving the same treatment.

### 4.1. Blood Parameters

The main blood parameters used to detect imbalances in the iron metabolism of mammals are hemoglobin, hematocrit, serum iron, and the iron-binding capacity [27]. Only the combination of several of these blood parameters and additional data on the performance and health of the animal enables a comprehensive evaluation of their iron status and its effect on their physical well-being [4,28].

The hematocrit is the volume percentage of erythrocytes in the whole blood. Since the synthesis of erythrocytes directly depends on the iron supply, erythrocyte production and, consequently, the hematocrit decreases when iron supply is limited [29,30]. The treatments in our study did not differ until day 14 post natum. Consequently, hematocrit in piglet blood also did not differ and was 24.1%–24.2% and 34.1%–35.0% by volume on day 3 and 14 post natum, respectively. The hematocrit level observed on day 14 post natum is similar to reports by Novais et al. [11], who found 34.6% hematocrit in piglet blood on the 8th day of life, after injecting 200 mg iron dextran on the third day of life. However, treatments Iron2 and Iron3 directly translated into significantly increased hematocrit levels, resulting in the significantly highest value in Iron3 (41.2%) and the significantly lowest value in Iron1 (31.6%) on day 28 post natum. This agrees with reports from Jolliff and Mahan [15], who found higher hematocrit values at weaning when piglets received an additional 100 mg Fe on their 10th day of life as compared to only a single injection of 200 mg Fe on their third day of life. While hematocrit in the treatments with two and three iron injections increased significantly until day 21 and stayed the same or even increased further on day 28, hematocrit in Iron1 already declined between day 21 and 28 post natum, indicating the short-term effect of a single iron injection. The negative effects of iron deficiency depend on frequency, duration and severity of the inadequate iron supply, and there are three categories of iron deficiency: pre-latent (iron store deficit), latent (decreased serum iron) and manifest (low hemoglobin and hematocrit concentration) iron deficiency [28]. The interpretation of hematocrit levels in piglets’ blood with regard to iron deficiency varies slightly between authors, with Bollwahn et al. [4] defining a hematocrit of at least 35% as normal for 28-day-old piglets. A hematocrit level lower than 34% for one- to six-week-old piglets [31] or lower than 33% for suckling piglets [32] indicates a manifest iron deficiency. Compared with these reference values, all piglets participating in our study were within the range interpreted as normal on day 14 and 21 post natum. On day 28 post natum, however, piglets assigned to Iron1 can be classified as iron deficient according to all above-mentioned recommendations. It can be assumed that these animals were only able to reach a physiologically normal hematocrit level when they started to take in a considerable amount of solid feed. The usual practice of supplementing iron once, therefore, seems to lead to a period of manifest iron deficiency. Treatments Iron2 and Iron3, on the other hand, resulted in hematocrit values within the range considered normal on day 14, 21, and 28 post natum.

Another blood parameter illustrating iron supply is serum iron, which is the concentration of iron bound to transferrin, the glycoprotein responsible for iron transport [29]. In a situation of iron deficiency, the iron stores (ferritin, hemosiderin) are emptied first (pre-latent deficiency); if the deficiency continues, serum iron level decreases (latent deficiency). Parallel to the development of hematocrit values, serum iron did not differ until day 14 post natum. Following the second iron injection, piglets assigned to Iron2 and Iron3 showed significantly higher serum iron concentrations on day 21 post natum than Iron1 piglets. While the single iron injection in Iron1 resulted in decreasing serum iron concentrations after day 14, an increase until day 21 post natum was documented for treatment Iron2. However, serum iron concentrations in Iron2 decreased between day 21 and day 28 post natum. Treatment Iron3 led to a continued increase, resulting in the significantly highest serum iron concentration on day 28 post natum. Literature sources related to iron supply in piglets differ in their statements on which serum iron content indicates an iron deficiency. In 1976, Pfau et al. interpreted serum iron concentrations lower than 26.86 µmol L^−1^ as latent iron deficiency in piglets, and lower than 17.91 µmol L^−1^ as indicating a manifest deficiency [31]. In 1983, Bollwahn et al. set the threshold of latent iron deficiency at a much lower level of 16.12 µmol L^−1^ [4]. The most recent recommendations of Heinritzi and Plonait define latent iron deficiency at serum iron concentrations lower than 21 µmol L^−1^, which is between the above-mentioned recommendations [32]. On day 14 post natum, serum iron concentrations of all piglets in our experiment indicated a latent iron deficiency according to Heinritzi and Plonait [32], and Iron2 and Iron3 piglet could be classified as manifest iron deficient according to Pfau et al. [31]. The optimum level of >26.87 µmol L^−1^ serum iron recommended by Pfau et al. [31] was never reached throughout the experimental period; only piglets assigned to Iron3 came close on day 28 post natum.

Summarizing our findings on the effect of iron supplementation on the iron status of piglets at our experimental farm, a single iron injection on day 3 led to both hematocrit level and serum iron concentration indicating latent to manifest iron deficiency until day 28 post natum. On day 14 post natum, however, hematocrit could be considered as normal. The treatments with two or three iron injections resulted in hematocrit levels within the normal range, and serum iron concentrations mostly indicated sufficient iron supply as well.

### 4.2. Performance and Health of Piglets

On organic pig farms with suckling periods of at least 40 days, the transition to solid feed happens later than on conventional farms, where piglets are usually weaned at an age of 21–28 days. In accordance with previous observations at our organically certified experimental station [33], we found that feed consumption reached a level of 100 g feed per piglet and day during the 5th week of life, an age at which conventionally reared piglets are already weaned. The last blood samples were taken on day 28, which means that iron intake from feed cannot have been considerable until then, especially because very young pigs tend to play with feed more than they eat it. Only during weeks 5–7 did feed consumption start to rise, resulting in an increasing iron supply from feed. However, because of the experimental design, it was not possible to separate between the treatments when documenting feed consumption. Therefore, it cannot be ruled out that some piglets compensated for inadequate iron supply with increased feed consumption.

The importance of adequate iron supply for unimpaired growth of piglets has been known for a long time [34,35], but results from studies on iron supplementation differ. Bruininx et al. compared a single iron injection of 200 mg iron dextran with two injections on day 3 and on day 21 post natum under the conditions of a conventional four-week suckling period and found no differences in the body weight of piglets until five weeks after weaning [16]. Murphy et al. also found no effect of a second iron injection on weight gain [17]. On the other hand, both Kamphues et al. and Haugegaard et al. observed a positive effect on daily weight gain of piglets when giving an additional 200 mg iron dextran at 3 weeks of age, even though piglets were already weaned at the age of 28 and 34 days, respectively [18,19]. Also, Bhattarai and Nielsen and Ettle et al. found that a considerable iron deficit resulted in the lower weight gain of piglets [29,36]. In our study, no difference in piglet growth was found until day 28 post natum, which agrees with reports from the above-mentioned results from Bruininx et al. and Murphy et al. [16,17]. However, the longer suckling period required in organic pig husbandry led to a significant effect of iron supplementation on piglet growth until weaning, which is in accordance with Kamphues et al. and Haugegaard et al. [18,19]. Treatments Iron2 and Iron3 did not differ from each other, but Iron1 resulted in significantly slower growth and consequently lighter piglets at weaning (Iron1: 11.5 kg; Iron2: 13.1 kg; Iron3: 13.1 kg).

The average daily weight gains found in treatments Iron2 and Iron3 were 274 and 276 g, respectively. This level of growth is common for our experimental pig herd [33], but lower than daily weight gains of 280 and 340 g during a suckling period of 5 and 7 weeks reported by Andersen et al. [37]. Previous studies on the iron metabolism of piglets have found negative effects of iron deficiency on blood cell count [8,19], immune defense and pre-weaning mortality [38,39]. Also, higher susceptibility to infectious diseases and disorders of the alimentary tract has been observed [40,41].

Although blood analysis of the piglets led to the conclusion that a single iron injection on day 3 post natum resulted in latent to manifest iron deficiency, no differences in the number of medical treatments and mortality during the experimental period could be observed.

Mortality was generally low, and amounted to 3.1%, 3.0% and 5.4% of piglets in treatments Iron1, Iron2, and Iron3, respectively. Prunier et al. reported a considerably higher mortality of 13.0%–25.5% on organic pig farms in eight European countries, which can be explained by the fact that their report includes early losses during the first days of life, while our experimental period lasted from day 3 post natum until weaning [1].

To summarize our observations, a single iron injection was not enough to ensure adequate iron supply and unimpaired growth in suckling piglets on our experimental farm. Considering that a single iron injection is the usual practice on organic pig farms, it seems that inadequate iron supply for suckling piglets is a problem that requires attention. Based on the differences in iron status and growth between the piglets receiving iron once or two or three times, additional iron supplementation seems highly recommendable. One option is a second (or third) injection as in our study. Oral application could be another option but would require a change in the regulations for organic agriculture.

## Figures and Tables

**Table 1:** Ingredients and nutrient composition of diets for piglets receiving different frequencies of iron supplementation, and their mother sows. One bulk sample of each feed mixture was analyzed, and values are given on a dry matter basis.

	Sows		Piglets
	Gestation	Lactation	Starter Feed
Ingredients, g kg^−1^			
Triticale	362	540	490
Barley	225		
Peas	63	140	115
Faba beans	130	155	153
Soybean cake		65	100
Rapeseed cake	15	45	45
Wheat bran	170		
Skimmed milk powder		10	50
Sunflower oil		8	10
Citric acid	5	7	7
Mineral mix *	30	30	30
Nutrient composition, g kg^−1^			
Dry matter	849	868	877
Crude protein	149	188	171
Ether extract	24.2	28.5	31.2
Crude fiber	43.3	53.7	40.0
Ash	52.2	51.4	50.6
Starch	514	493	506
Sugar	46.6	59.9	69.4
ME, MJ kg^−1^ **	14.7	14.6	15.1
Lysine	8.1	11.4	10.6
Methionine + cysteine	4.3	5.3	5.0
Threonine	5.3	6.8	6.7
Tryptophan	1.5	1.7	1.6
Calcium	8.2	8.1	7.4
Phosphorus	5.3	4.7	4.6
Iron, mg kg^−1^ ***	87	83	81

* Mineral mix for sows provided the following amounts per kilogram of feed: 6.3 g Ca, 1.8 g P, 2.1 g Na, 0.45 g Mg, 12,000 IU vitamin A, 2000 IU vitamin D3, 105 mg vitamin E, 75 mg Zn (zinc oxide), 45 mg Fe (iron sulphate), 45 mg Mn (manganese oxide), 12 mg Cu (copper sulphate), 1.5 mg I (calcium iodide), and 0.42 mg Se (sodium selenite). Mineral mix for piglets provided the following amounts per kilogram of feed: 6.9 g Ca, 2.25 g P, 1.65 g Na, 0.45 g Mg, 10,000 IU vitamin A, 1710 IU vitamin D3, 120 mg vitamin E, 75 mg Zn (zinc oxide), 30 mg Fe (iron sulphate), 45 mg Mn (manganese oxide), 15 mg Cu (copper sulphate), 1.5 mg I (calcium iodide), and 0.42 mg Se (sodium selenite). ** ME: metabolizable energy, calculated according to Society for Nutritional Physiology (GfE) [23]. *** Iron concentration was calculated based on literature values [22].

**Table 2:** Effect of supplementing iron by subcutaneous injection to organically reared piglets on their third day of life only (Iron1), on day 3 and 14 (Iron2) and on day 3, 14 and 21 (Iron3) on hematocrit content in blood (% by volume). Results are given as least square estimates ± standard errors of the means (SEMs) of the interaction treatment * day, and *p* values for the effects of treatment, day and treatment × day, and were calculated using SAS PROC MIXED.

	Iron Supplementation			*p* Values		
	Iron1	Iron2	Iron3	Treatment	Day	Treatment × Day
	*n* = 96	*n* = 99	*n* = 93			
Sampling day (post natum)						
3	24.2 ^A^ ± 0.55	24.1 ^A^ ± 0.55	24.1 ^A^ ± 0.56	<0.001	<0.001	<0.001
14	35.0 ^C^ ± 0.56	34.1 ^B^ ± 0.56	34.7 ^B^ ± 0.58			
21	34.2 ^a,C^ ± 0.58	39.1 ^b,C^ ± 0.57	39.1 ^b,C^ ± 0.58			
28	31.6 ^a,B^ ± 0.56	39.4 ^b,C^ ± 0.55	41.2 ^b,D^ ± 0.57			

^a,b^ Values within a row with different superscripts differ significantly at *p* < 0.05; ^A,B,C,D^ Values within a column with different superscripts differ significantly at *p* < 0.05.

**Table 3:** Effect of supplementing iron by subcutaneous injection to organically reared piglets on their third day of life only (Iron1), on day 3 and 14 (Iron2) and on day 3, 14 and 21 (Iron3) on serum iron content (µmol L^−1^). Results are given as least square estimates ± SEMs of the interaction treatment * day and *p* values for the effects of treatment, day and treatment * day and were calculated using SAS PROC MIXED.

	Iron Supplementation			*p* Values		
	Iron1	Iron2	Iron3	Treatment	Day	Treatment × Day
	*n* = 96	*n* = 99	*n* = 93			
Sampling day (post natum)						
3	5.2 ^A^ ± 0.80	4.6 ^A^ ± 0.79	4.6 ^A^ ± 0.81	<0.001	<0.001	<0.001
14	18.5 ^B^ ± 0.81	16.4 ^B^ ± 0.81	15.5 ^B^ ± 0.83			
21	8.3 ^a,A^ ± 0.83	23.6 ^b,C^ ± 0.82	25.4 ^b,C^ ± 0.83			
28	6.3 ^a,A^ ± 0.81	20.5 ^b,C^ ± 0.79	26.6 ^c,C^ ± 0.83			

^a,b,c^ Values within a row with different superscripts differ significantly at *p* < 0.05; ^A,B,C^ Values within a column with different superscripts differ significantly at *p* < 0.05.

**Table 4:** Effect of supplementing iron by subcutaneous injection to organically reared piglets on their third day of life only (Iron1), on day 3 and 14 (Iron2) and on day 3, 14 and 21 (Iron3) on body weight and daily weight gain until weaning. Results are given as least square estimates ± SEMs of the interaction treatment * day for body weight and least square estimates ± SEMs of the effect of treatment for daily weight gain, and *p* values for the effects of treatment, day and treatment * day. Analysis was carried out using SAS PROC MIXED.

	Iron Supplementation			*p* Values		
	Iron1	Iron2	Iron3	Treatment	Day	Treatment × Day
	*n* = 96	*n* = 99	*n* = 93			
Body weight on day (post natum), kg						
3	1.89 ± 0.17	1.89 ± 0.17	1.89 ± 0.17	<0.001	<0.001	<0.001
14	4.6 ± 0.17	4.6 ± 0.17	4.7 ± 0.17			
21	6.3 ± 0.17	6.5 ± 0.17	6.5 ± 0.17			
28	7.9 ± 0.17	8.1 ± 0.17	8.0 ± 0.17			
Weaning	11.6 ^a^ ± 0.17	13.1 ^b^ ±0.17	13.1 ^b^ ± 0.18			
Daily weight gain until weaning, g						
Day 0–42	240 ^a^ ± 8.0	274 ^b^ ± 7.9	275 ^b^ ± 8.1	<0.001		

^a,b^ Values within a row with different superscripts differ significantly at *p* < 0.05.

**Table 5:** Incidence of medical treatments and mortality of organically reared piglets receiving iron supplementation by subcutaneous injection on their third day of life only (Iron1), on day 3 and 14 (Iron2) and on day 3, 14 and 21 (Iron3).

	Iron Supplementation		
	Iron1	Iron2	Iron3
	*n* = 96	*n* = 99	*n* = 93
Medical treatments			
Day 3–14	4	3	4
Day 14—weaning	4	4	1
Mortality			
Day 3–14	2	1	4
Day 14—weaning	2	2	2

## Supplementary Materials

The following is available online at https://www.mdpi.com/2076-2615/9/9/651/s1, Table S1: Piglet growth and iron status when supplementing iron in different frequencies.
